# Therapy of Pulmonary Tuberculosis

**Published:** 1911-08

**Authors:** Louis C. Rouglin

**Affiliations:** Atlanta, Ga.


### THERAPY OF PULMONARY TUBERCULOSIS.

By Dr. Louis C. Rouglin, Atlanta, Ga.

Of all chronic diseases tuberculosis is most amenable to treatment, but, the most difficult disease to treat. The cure depends largely upon the early recognition of the disease, the promptness in the application of treatment, the intelligence of the physician applying same, and upon the intelligent co-operation of the patient receiving treatment. There is probably no disease which is more mistreated, for the very good reason that there is no disease more difficult to treat.

Before beginning treatment the physician must make as thorough an examination as possible, not only of the lungs, but, of the entire condition of the patient. He must study tthe temperament, the nervous condition, previous environments, habits, education, and vocation of the patient, and recognize that this varies in different individuals. He must prescribe such remedies, diet, and give such instructions as will suit the different patients. This disease requires for its successful treatment the strictest individualization. You must adapt remedies to suit the patient and his disease. *You can not make the patient or the disease adapt themselevs to your remedies.*

The physician must have the confidence and co-operation of the patient. The disease does not run a smooth, even course under the best conditions. The patient has times when he is not feeling well, in fact, his condition may seem apparently much worse. The physician must be able to satisfy his patient, explain the cause of the changes occurring, make his contented and willing to continue the fight. You must remember that it is not so easy for a patient to understand the reason for your various orders. He does not know the pathology of the disease, does not understand the chronicity of its course, he tires, and it is difficult to remain good, and co-operate for a long time, and be contented, even when one knows it is necessary for a cure.

On the other hand, you must also be able to keep the patient on guard when he is getting well. He must be made to realize that this is a dangerous time; you must prevent over-confidence without depressing the patient. The patient must be *willing to let you guide him* on all occasions, circumstances, and conditions. *You can not make him do it.* I have seen in sanatoria patients who were getting along well, consider the santorium a place of amusement and pleasure, instead of a place for the care of tuberculosis. It is almost impossible to individualize in private institutions. Such places must have a system—usually such a system is based on profit and business principle. All cases can not adapt themselves to such a system. To some cases such a particular system is absolutely injurious.

It is not the purpose of this paper to advocate any special specific medication. The use of vaccines and tuberculins in selected cases is of utmost value and benefit to the patient, but it is not applicable to all cases; and their administration in selected cases must be governed by the individual conditions that may arise during the administration. Used indiscriminately, they are bound to produce a great deal of harm, and little, if any, good. Neither it is my intention to recommend a long list of drugs to pour down the patient's throat. We have graduated from the stage of vaunted specific, which are not specifics, and also are rapidly passing out of the stage of expectorants, tonics, emulsions, etc., to the rational management of the disease by means of hygiene, rest, diet, open air, and scientific guiding.

Rest is essential for repair of tuberculous tissue. It reduces night sweats and temperature, its prevents exhaustion, prostration and dyspnoea, it helps the cough, and diminishes the danger of hemorrhage. In moderately advanced cases rest is of more importance than open air. The rest should be *complete rest—mental* and *physical,* with the actual relaxation of the patient in bed for 24 hours. A temperature of 99.5 degrees contraindicates the permission of any form of exercises—especially walking; an afternoon temperature of 100 degrees, despite the absence of fever earlier in the day, demands a restriction of all physical effort; while a temperature of 101.5 degrees and over demands the recumbent position in bed indefinitely until the fever has materially abated. As a whole, fever, weak, or rapid pulse, is a contra-indication for any physical exercise, and when exercise is resumed, the utmost precaution should be taken against fatigue.

Pure, fresh air is essential, and the patient should be encouraged to stay out in the open air all the time, proper care being taken to avoid catching cold, or chilling the body. On the whole, the temperature of the air is of no importance—tubercular patients are only affected by rapid changes, or sudden falls of temperature, and proper precautions should be taken for these unavoidable conditions. For night air the ordinary sleeping room provided with a Dr. Knopf window tent has many advantages, and is in my opinion far superior to the ordinary shacks, or tents.

In cases where no lesion can be found, but where there is a tendency to tuberculosis as shown by history, chest formation, or exposure to infection, and in absence of fever, much good can be derived from open air exercises, accompanied by training of respiratory powers. In these cases the rule of Brehmer must be constantly borne in mind, "The healthy man sits down because he is tired, the consumptive should sit down so as not to become tired."

Patients should be impressed with the fact that all overexertion is poison, and that their feelings should be their guide at all times. Perspiration, palpitation, acceleration of pulse, rise of temperature, feeling of weakness, discomfort and headache are all signs of having overstepped the limit.

The patient should from the outset be impressed with the importance of diet in this disease. Directions should not be given in a general way, but should be specific, covering both the articles to be eaten and those to be avoided, the time for taking the food and the amount to be taken should be carefully outlined. These points vary with different patients, but each case must be studied individually in order to attain the best success. The nutrition of a patient is a reliable guide as to the progress of the disease. If the patient takes sufficient nutritious food, is digesting it and gaining in weight, the prognosis is good. If the reverse is the case, the prognosis is bad. A persistent inability to digest food is one of the most unfavorable symptoms—care must be taken to avoid disturbing the digestive system. The appetite is not a good guide to the amount of food to be taken. In most cases more food can be digested than the appetite demands. The desire of the patient should be consulted as far as possible; the nationality, character, and previous customs as regard to diet must be considered. Each case must be studied individually and diet prescribed to suit each individual case and taste. There can be no successful routine diet for all cases, even of the same class. The patient should be instructed to rest at least half an hour before and after each meal, and a good mouth wash prescribed to rinse the mouth before and after eating.

The intelligent use of drugs as remedies to meet the varying needs, requirements, and complications of tuberculous individuals is not only eminently proper, but absolutely essential. The extent to which the general practititoner and laity have been impressed with the supposed disadvantage of drugs for the consumptive has produced an unreasonable and unjustified prejudice against its employment, and is the cause of much embarrassment to the intelligent physician who seeks to utilize the beneficial effects of judicious medication in order to control untoward symptoms of hemorrhage, the restoration of the various digestive disturbances, disorders of elimination, and the alleviation of the various disturbed functions are often wonderfully facilitated by the judicious and selective choice of drugs—and to deny their usefulness in the hands of discriminative, conscientious, resourceful physicians is as unwarranted as to repudiate the value of drugs in any disease. For the general condition the indications for drug therapy are few, and in comparison to the other measures is relatively unimportant. In selected cases, however, the intelligent use of drugs is of some value.

Creosote and its derivatives have been the most used as well as the most abused drugs in this disease. It has no specific action upon tuberculosis, but in selected cases I have found it valuable in small doses minims 3 to 5, for the secondary infections, such as simple bronchitis, also for its stimulating effect upon the bronchial mucous membrane, while in hyper-pyrexia with nausea or for analgesia in complicating neuritis and intercostal neuralgia I have found icthyol in 5 grain doses to be of decided benefit.

Icthyol given in doses of 10 drops mixed with an equal amount of pure olive oil continued for some time is of utmost value. It is well borne by the stomach, it loosens and reduces expectoration, increases the appetite and seems to have a fine effect upon the nervous system. To overcome its disagreeable taste, I prescribe it in elastic capsules. It also seems to have a beneficial effect in diarrhoeal conditions.

Strychnine alone or combined with arsenic is of great advantage as a general stimulant. It helps the lowered blood tension and jaded heart; it should not be given in too large doses for a long time, and especially the effect of the arsenic should be watched carefully. For general purposes, in the absence of contra-indications the administration of gr. 1-60 of strychnine with gr. 1-60 arsenic or, *one* to *three* drops of Fowler's solution will increase the appetite and stimulate the general functional activities.

The use of easily digested fats and oils, such as are usually found in the market is of advantage in a certain class of patients. Much care should be exercised in their employment on account of their tendency to retard digestion. They should not be given to patients with fever or gastric derangements. As a general rule, they are better borne in winter than in summer. Their greatest usefulness is found in the treatment of individuals who can not, for financial reasons, obtain the proper amount of nutritious diet. But where the patient is able to supply himself with plenty of good cream, butter and eggs, the use of emulsions of fats and oils is superfluous, and in such cases may even be considered injurious.

Inhalations and sprays are of value only for the local catarrhal affections, such as laryngitis, pharyngitis, and possibly simple bronchitits. It has not effect upon the disease; the routine inhalation of oxygen is absolutely of no value. Oxygen itself has no effect in the disease; the haemoglobin absorbs all the oxygen it can hold in combination from the air, and super-oxygenated gases increase only the oxygen in the serum. Ozone is an indicator of pure, fresh air, *but ozone itself is not pure, fresh air.* It is an irritant to the respiratory mucous membranes, and more injurious when it is absorbed.

In conclusion, I desire to impress the fact that in tuberculosis there is no single element, neither air, diet, vaccines, nor drugs, which affords any guarantee of success in the cure of this disease, but that it requires the satisfying of the demand of all the physiological and hygienic requirements of the organism. The physician must devote himself to the task with unceasing patience, and strive with iron determination towards the goal. In no disease does the physician's personality and tact as, well as the intelligence of the patient, have a greater bearing on the favorable outcome, and in no disease is the saying of Alexander Pope:

"Be not the first by whom the new is tried
Nor yet the last to lay the old aside,"

more truly applicable than in the therapy of Pulmonary Tuberculosis.

757-758 Candler Annex.

